# Relevance of Plasma Obestatin and Early Arteriosclerosis in Patients with Type 2 Diabetes Mellitus

**DOI:** 10.1155/2013/563919

**Published:** 2013-11-17

**Authors:** Peng-ying Gu, Dong-mei Kang, Wei-dong Wang, Yan Chen, Zhi-hong Zhao, Hui Zheng, Shan-dong Ye

**Affiliations:** ^1^Department of Geriatric Medicine, Anhui Provincial Hospital Affiliated to Anhui Medical University, Hefei, Anhui 230001, China; ^2^Department of Endocrine Laboratory, Anhui Provincial Hospital Affiliated to Anhui Medical University, Hefei, Anhui 230001, China; ^3^Department of Ultrasonic Medicine, Anhui Provincial Hospital Affiliated to Anhui Medical University, Hefei, Anhui 230001, China; ^4^Department of Clinical Laboratory, Anhui Provincial Hospital Affiliated to Anhui Medical University, Hefei, Anhui 230001, China; ^5^Department of Endocrinology, Anhui Provincial Hospital Affiliated to Anhui Medical University, Hefei, Anhui 230001, China

## Abstract

We investigated the correlation between obestatin and metabolic parameters and carotid intima-media thickness (IMT) in plasma of patients with type 2 diabetes mellitus (T2DM). We collected 103 patients aged from 60 to 83 years (69.26 ± 5.83 years) form January, 2007 to May, 2009. All patients were divided into normal glucose tolerance (NGT), impaired glucose tolerance (IGT), and T2DM according to the oral glucose tolerance test (OGTT). We found that higher levels of fasting insulin (Fins), fasting blood glucose, 2 h OGTT glucose, homeostasis model assessment of insulin resistance (HOMA-IR), low density lipoprotein cholesterol, glycated haemoglobin, and C-reactive protein (CRP), as well as lower obestatin level and higher intima-media thickness level (IMT), existed in T2DM group compared with NGT group and IGT group (*P* < 0.01). Also, obestatin level was independently associated with HOMA-IR and CRP, while IMT level was independently associated with HOMA-IR, triglyceride, Fins, and obestatin (*P* < 0.01), based on stepwise multiple regression analysis. Therefore, we deduced that the low level of plasma obestatin might be related to early arteriosclerosis in patients with T2DM via increasing IMT level, and elevated plasma obestatin levels might protect T2DM patients against carotid atherosclerosis to some extent.

## 1. Introduction

Type 2 diabetes mellitus (T2DM) is related to a significant increase in the risk of atherosclerosis [1]. In the past several years, clinical studies have shown that high mortality in patients with T2DM is due to cardiovascular disease [2]. Besides, T2DM often coexists with dyslipidemia, coronary disease, hypertension, and visceral obesity [3, 4]. As is well known, cardiometabolic risk factors play important roles in pathophysiology of arteriosclerosis and diabetes, including hormones in the appetite and body weight regulation, adipokine (leptin, resistin, and adiponectin), ghrelin, and obestatin [5, 6]. 

With the development of blood glucose-lowering medications, such as lifestyle-directed interventions, insulin, sulfonylureas, and metformin, the number of treatment options available for T2DM has increased recently [7]. Besides, rosiglitazone maleate targets insulin resistance to enhance the synthesis of glucose transporters and activate adipocyte differentiation, while metformin hydrochloride can promote the lowering glucose by reducing hepatic glucose production and enhance the gluconeogenesis; by increasing peripheral glucose uptake, the combination of two drugs is effective and safe in reducing hyperglycemia in patients with T2DM [8]. Although numerous reviews on the management of T2DM have been published in recent years [9, 10], a clear pathway of therapy has not been confirmed.

Obestatin, a 23-amino acid peptide derived from the ghrelin precursor protein, is a risk factor for cardiovascular disease [11]. In previous studies, obestatin was found correlated with intima-media thickness (IMT), which is regarded as a biomarker of early arteriosclerosis, suggesting that obestatin plays a positive role in inhibiting carotid atherosclerosis at the early stage [12]. Obestatin also could improve myocardial function and decrease apoptosis of cardiomyocytes in isolated rat heart, which were regulated by specific obestatin receptors on cardiac cells or via activation of reperfusion injury salvage kinase (RISK) pathways [13]. Currently, obestatin has been identified to be associated with insulin resistance metabolic dysfunctions, suppressing food intake, jejunal contraction, and body weight gain [14]. For instance, obestatin inhibits secretion of pancreas polypeptide and promotes generation of pancreatic juice enzymes through a vagal pathway [15, 16]. Furthermore, the G protein-coupled orphan receptor (GPR39), which is regarded as receptor of obestatin, is decreased in adipose tissue of obese patients with T2DM [17, 18].

Although previous study have demonstrated that diabetes patients with fasting plasma glucose above 7.0 mM/L have a higher risk of cardiovascular death [19], studies focus on the relationship between obestatin and arteriosclerosis in patients with T2DM are insufficient. Therefore, in our study, we examined the plasma level of obestatin and metabolic parameters to investigate the relationship among obestatin, arteriosclerosis, and T2DM.

## 2. Materials and Methods

### 2.1. Subjects

All persons have given their informed consent prior to their inclusion in the study, and all human studies have been approved by the China Ethics Committee and performed in accordance with the ethical standards. One hundred and three patients aged, which undergone physical examination and hospitalization from 60 to 83 years (69.26 ± 5.83 years), were selected from the Clinic for Retired Veteran Cadres, Anhui Provincial Hospital Affiliated to Anhui Medical University from January, 2007 to May, 2009. 

After consulting the detail of medical history and physical examination, determining thyroid function, biochemical indicators, and endocrine hormone; subjects with stress hyperglycemia, autoimmune diseases, thyroid dysfunction, acromegaly, hypercortisolism, liver or renal insufficiency, malignant tumor, or continuous use of glucocorticoid, diuretic, niacin, or indomethacin were excluded. All patients, without diabetic complications, without factors of secondary rise in blood sugar, without diabetic vascular complications, and without administered drugs for gastrointestinal system within three months were included in this research. Among patients, 40 subjects had history of high blood pressure, the disease course was 3~10 months, and the person who was given drugs with the effects on insulin-resistance was excluded; totally 59 persons were diagnosed with hyperlipemia, the disease course was 0~7 years, there was no long-term users of lipid-lowering drugs; 10 subjects were diagnosed with coronary heart disease with arteriography examination; and 30 subjects represented myocardial ischemia with electrocardiograph. All subjects received oral glucose tolerance test (OGTT) and were divided into three groups, normal glucose tolerance (NGT), impaired glucose tolerance (IGT), and T2DM, according to the diagnostic criteria published by the World Health Organization (WHO), in 1999. The T2DM patients were newly diagnosed.

### 2.2. Methods

General information of the subjects was recorded including gender, age, height, weight, and blood pressure (BP). Body mass index (BMI) was calculated as weight divided by height squared (kg/m^2^). BP was measured twice (5 min interval between the two measurements) with an accurate mercury sphygmomanometer after a 5-min rest, and the average value was calculated. Venous blood was collected after overnight fasting. The contents of fasting insulin (Fins) were measured with an Abbott bichromatic analyzer (Abbott Labs, USA). Fasting plasma insulin levels were measured with a commercial radioimmunoassay (Diagnostic Products, USA). Levels of Fins and FBG were measured at 120 min after glucose load (OGTT-2 h). Homeostasis model assessment of insulin resistance (HOMA-IR = Fins × FPG/22.5) was determined. Glycosylated haemoglobin (HbA1c) was measured by isoelectric focusing. Fasting plasma lipid parameters were detected with an automatic enzymatic analyzer (Hitachi 7600ISE-020, Japan) including total cholesterol (TC), triglyceride (TG), high-density lipoprotein cholesterol (HDL-c), and low-density lipoprotein cholesterol (HDL-c). Apolipoprotein (ApoA1 and ApoB) was measured by nephelometric assay (Diasys, German). C-reactive protein (CRP) was assayed with ELISA. Obestatin was assayed using enzyme immunoassay kits (lot 09041254, R&D systems, USA). IMT of carotid artery was blindly scanned three times by a B-mode color Doppler ultrasound with a frequency of 7.5 to 10 MHz. IMT was electronically measured three times at the far wall of the distal common carotid arteries, about 1 cm from the carotid bifurcation. The average values of bilateral arteries were obtained.

### 2.3. Statistical Analysis

All statistical analyses were performed on SPSS 13.0 (SPSS Inc, Chicago, USA). Normal distribution was assessed by independent sample *t*-test between the two groups. Differences between multigroups were compared using a one-way ANOVA. Data expressed as mean ± SD. Nonnormal distribution was performed with Kruskal-Wallis test. The relationships between two variables were assessed with Pearson and rank correlation. Stepwise regression was used to explore the correlation of obestatin and IMT. 

## 3. Results

### 3.1. Clinical and Biochemical Characteristics

The metabolic indexes of the patients in three groups were listed in Table 1. There were no differences of age, diastolic blood pressure (DBP), systolic blood pressure (SBP), and BMI levels between the three groups (*P* > 0.05). Differences of concentrations of biochemical parameters in the three groups were significant (*P* < 0.01). Increased levels of Fins, FPG, OGTT-2 h (2hPG), HOMA-IR, HbA1c, LDL-C, and CRP were detected in T2DM group compared with the other two groups (*P* < 0.01). Compared to NGT group, levels of TC, TG, and comparing with NGT group, higher levels of TC and TG were observed in T2DM group, as well as lower level of HDL-C (*P* < 0.01).

### 3.2. Levels of Obestatin and IMT

**Figure 1:**
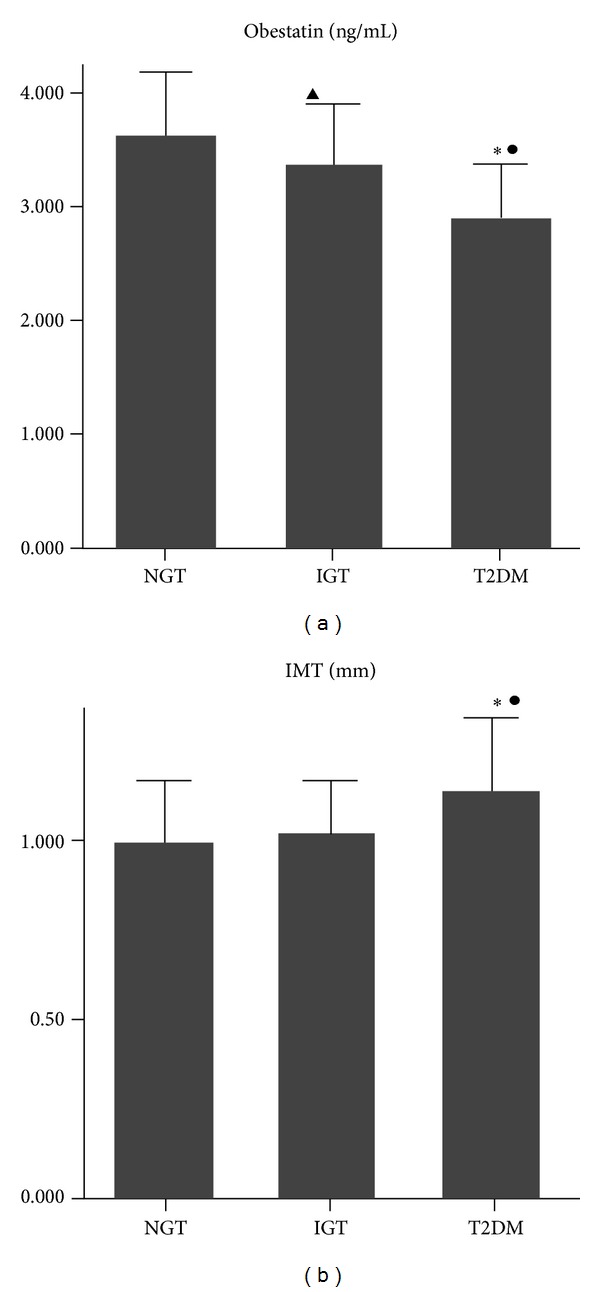
Levels of obestatin (a) and IMT (b) in patients of NGT, IGT, and T2DM groups. ▲: *P* < 0.05 compared with NGT group (the control group), ∗: *P* < 0.01 compared with NGT group, ●: *P* < 0.01 compared with IGT group, and IMT: intima-media thickness.

Patients with T2DM had a lower level of obestatin by comparing with the NGT and IGT groups (*P* < 0.01, Figure 1(a)), and the obestatin level in patients of IGT group was lower than that in the NGT group (*P* < 0.05). Increased levels of IMT were detected in T2DM group compared to NGT and IGT groups (*P* < 0.01, Figure 1(b)). However, no significant difference of IMT levels was observed between IGT group and NGT group.

### 3.3. Correlations between Obestatin and IMT

As shown in Table 2, the obestatin level was significantly correlated with BMI (*r* = −0.365, *P* = 0.004), Fins (*r* = −0.269, *P* = 0.036), HOMA-IR (*r* = −0.345, *P* = 0.006), FBG (*r* = −0.286, *P* = 0.025), HbA1c (*r* = −0.310, *P* = 0.015), TG (*r* = −0.345, *P* = 0.007), HDL-C (*r* = 0.261, *P* = 0.008), CRP (*r* = −0.524, *P* = 0.000), and IMT (*r* = −0.269, *P* = 0.036). Through stepwise multiple regression analysis, we found that obestatin level was independently associated with HOMA-IR and CRP (obestatin = 3.926 − 0.053 × HOMA − IR − 0.270 × CRP), residuals of values conformed to normal distribution.

IMT level was found to correlate positively with Fins (*r* = 0.269, *P* = 0.036), FBG (*r* = 0.238, *P* = 0.048), 2hPG (*r* = 0.278, *P* = 0.030), HOMA-IR (*r* = 0.288, *P* = 0.024), TG (*r* = 0.361, *P* = 0.004), and CRP (*r* = 0.289, *P* = 0.024), as well as negatively with obestatin (*r* = 0.269, *P* = 0.036) in simple regression analysis in the pooled data (Table 3). While the results of stepwise multiple regression analysis showed that IMT level was independently associated with HOMA-IR, TG, Fins, and obestatin (IMT = 1.233 + 0.044 × HOMA − IR + 0.054 × *TG* − 0.010 × Fins − 0.095 × obestatin). Residuals of values conformed to normal distribution.

## 4. Discussion

In this study, in T2DM, IGT, and NGT three groups, significantly higher plasma levels of Fins, FBG, 2hPG, HOMA-IR, TC, TG, LDL-C, and CRP were detected in T2DM subjects compared with NGT subjects (*P* < 0.01), and only the level of Fins, FBG, 2hPG, HOMA-IR, HbA1c, CRP, and LDL-C in NGT patients were obviously different with IGT patients. Significantly decreased obestatin level was also found in patients with T2DM by comparing with NGT patients and IGT patients, as well as IMT level comparing with NGT patients. Besides, obestatin was independently correlated with IMT plasma levels in the diabetic patients. Obestatin was considered as a nutritional marker reflecting insulin resistance [20], body adiposity, and a regulator of adipocyte metabolism [21, 22]. In previous studies, plasma obestatin level that was indicated was lower in patients with T2DM and IGR than in controls, and the multiple logistic regression analysis revealed obestatin to be independently associated with IGR and T2DM [23]. Meanwhile, the expression of GPR39, which was identified as the receptor for obestatin, displayed significantly lower levels in obese T2DM patients than in lean and obese normoglycaemic subjects; the mRNA expression levels of GPR39 was also negatively related to fasting glucose concentrations but represented a positive correlation to adiponectin mRNA expression levels; this suggested an involvement of obestatin signal pathway in glucose homeostasis and T2DM development [24] while St-Pierre et al. reported that normal and diabetic subjects display similar levels of circulating obestatin in fasting condition [25]. But in this study, plasma level of obestatin was decreased in diabetic patients. Thus, we supposed that lower level of obestatin could be a predictor for patients with T2DM, while the mechanisms of a reduction in obestatin levels in diabetic population are not quite clear. We supposed that the high level of obestatin may be a defense of plasma hyperglycemia. 

Carotid arterial intima-media thickness was used as a noninvasive surrogate end point to measure progression of atherosclerosis and is increasingly used as a surrogate marker due to its ability to predict future clinical cardiovascular end points [26, 27]. And increased IMT was considered not only a biomarker of arteriosclerosis in the early phases but also related with a clustering of risk factors for myocardial infarction [4, 28]. Previously, type 2 diabetes was associated with an 0.13 mm increase in IMT compared with control subjects [29]. And 6-month intensive lifestyle modification intervention in T2DM patients showed enhanced glycemic control and decreased progression of carotid IMT [30]. Increased common carotid artery IMT and plaque score were also associated with acute ischemic stroke in T2DM patients [31]. Moreover, the present study showed that IMT was significantly increased in patients with T2DM, and the plasma level of obestatin was correlated negatively to IMT; this suggested that high level obestatin might have a positive effect against carotid atherosclerosis. 

In addition, obestatin administration had been demonstrated to exert a beneficial effect against myocardial dysfunctions and cardiomyocyte apoptosis in a rat model [32, 33]. Obestatin also displays protective effect in cardiac function in humans, both in physiological and pathological situations. It is demonstrated that obestatin induces vascular relaxation by activating endothelium-dependent NO signaling [34]. Obestatin together with TNF-*α* treatment even decreased vascular cell adhesion molecule-1 expression and increase binding of LDL to macrophages, indicating the regulation of obestatin in the early atherogenic processes [35]. Hence, these evidences confirmed the effective protection of high level obestatin against the complication, such as atherosclerosis and myocardial infarction.

## 5. Conclusion

In conclusion, we have determined the levels of metabolic parameters in healthy people and patients with IGT or T2DM. Inverse correlation between obestatin and IMT has been demonstrated. The lower obestatin level in plasma might be an indicator of early arteriosclerosis. The increasing of plasma obestatin level might protect T2DM patients against carotid atherosclerosis complication to some extent. However, the limitations of this study existed including the small-scale of subjects with T2DM and the elderly age of enrolled subjects; the mechanism of obestatin protecting T2DM patients against arteriosclerosis in patients with T2DM needs to be investigated in long-term and large-scale studies.

## Figures and Tables

**Table 1:** Clinical and biochemical parameters of T2DM, IGT, and NGT groups.

Variables	NGT	IGT	T2DM
Number (men/women)	42 (29/13)	27 (20/7)	34 (22/12)
Age (y)	68.50 ± 5.80	70.22 ± 6.82	69.44 ± 5.00
SBP (mmHg)	132 ± 14	138 ± 14	136 ± 16
DBP (mm Hg)	79 ± 9	81 ± 7	81 ± 10
BMI (kg/m^2^)	23.85 ± 2.24	24.36 ± 2.95	24.93 ± 2.59
Fins (mU/L)	10.55 ± 6.35	12.80 ± 5.69	18.63 ± 7.11^∗∗▲▲^
FBG (mmol/L)	4.71 ± 0.65	5.12 ± 0.73*	6.14 ± 1.00^∗∗▲▲^
2hPG (mmol/L)	6.44 ± 0.94	9.40 ± 1.34**	14.25 ± 2.51^∗∗▲▲^
HbA1c (%)	4.79 ± 0.50	5.02 ± 0.50**	5.93 ± 0.47^∗∗▲▲^
HOMA-IR	2.27 ± 1.50	3.31 ± 1.56*	5.23 ± 2.35^∗∗▲▲^
TC (mmol/L)	4.57 ± 1.00	5.37 ± 1.06**	5.54 ± 1.00**
TG (mmol/L)	1.75 ± 1.54	2.08 ± 1.36	2.70 ± 0.96**
HDL-C (mmol/L)	1.35 ± 0.40	1.25 ± 0.52	1.10 ± 0.25**
LDL-C (mmol/L)	2.63 ± 0.74	3.11 ± 1.08**	3.83 ± 0.85^∗∗▲▲^
CRP (mg/L)	0.971 ± 0.464	1.340 ± 0.894	2.275 ± 0.968^∗∗▲▲^

Data were expressed as mean ± SD. *P < 0.05 compared with NGT group; **P < 0.01 compared with NGT group; ^▲^ *P* < 0.05 compared with IGT group; ^▲▲^ *P* < 0.05 compared with IGT group. SBP: systolic blood pressure; DBP: diastolic blood pressure; BMI: body mass index; Fins: fasting insulin; FBG: fasting blood glucose; 2hPG: 2 h OGTT glucose; HbA1c: glycated haemoglobin; HOMA-IR: homeostasis model assessment-insulin resistance index; TC: total cholesterol; TG: total triglyceride; HDL-C: high density lipoprotein cholesterol; LDL-C: low density lipoprotein cholesterol; CRP: C-reactive protein.

**Table 2:** Correlation analysis of obestatin and various metabolic parameters.

Variable	Simple		Multiple	
	Estimate	*P* value	Estimate	*P* value
Age	−0.107	0.412		
SBP	0.220	0.088		
DBP	−0.097	0.455		
BMI	−0.365	0.004		
FIns	−0.269	0.036		
FBG	−0.286	0.025		
2hPG	−0.243	0.060		
HOMA-IR	−0.345	0.006	−2.056	0.042
HbA1c	−0.310	0.015		
TC	−0.056	0.671		
TG	−0.345	0.007		
HDL-C	0.261	0.008		
LDL-C	−0.165	0.203		
CRP	−0.524	0.000	−4.353	0.000
IMT	−0.269	0.036		

SBP: systolic blood pressure; DBP: diastolic blood pressure; BMI: body mass index; Fins: fasting insulin; FBG: fasting blood glucose; 2hPG: 2 h OGTT glucose; HOMA-IR: homeostasis model assessment-insulin resistance index; HbA1c: glycated haemoglobin; TC: total cholesterol; TG: total triglyceride; HDL-C: high density lipoprotein cholesterol; LDL-C: low density lipoprotein cholesterol; CRP: C-reactive protein; IMT: intima-media thickness.

**Table 3:** Correlation analysis of variables associated with IMT in subjects studied.

Variable	Simple		Multiple	
	Estimate	*P* value	Estimate	*P* value
Age	−0.052	0.693		
SBP	0.085	0.512		
DBP	0.054	0.681		
BMI	0.007	0.959		
FIns	0.269	0.036	−2.852	0.006
FBG	0.238	0.048		
2hPG	0.278	0.030		
HOMA-IR	0.288	0.024	3.822	0.000
HbA1c	0.118	0.367		
TC	0.171	0.186		
TG	0.361	0.004	3.325	0.002
HDL-C	−0.009	0.945		
LDL-C	0.064	0.623		
CRP	0.289	0.024		
Obestatin	−0.269	0.036	−2.581	0.012

SBP: systolic blood pressure; DBP: diastolic blood pressure; BMI: body mass index; Fins: fasting insulin; FBG: fasting blood glucose; 2hPG: 2 h OGTT glucose; HOMA-IR: homeostasis model assessment-insulin resistance index; HbA1c: glycated haemoglobin; TC: total cholesterol; TG: total triglyceride; HDL-C: high density lipoprotein cholesterol; LDL-C: low density lipoprotein cholesterol; CRP: C-reactive protein.
